# First totally laparoscopic ALPPS procedure with selective hepatic artery clamping: Case report of a new technique

**DOI:** 10.1097/MD.0000000000004236

**Published:** 2016-07-22

**Authors:** Rodrigo C. Surjan, Fabio F. Makdissi, Tiago Basseres, Denise Leite, Luiz F. Charles, Regis O. Bezerra, Erik Schadde, Marcel Autran Machado

**Affiliations:** Department of Surgery, University of Sao Paulo, Sao Paulo, Brazil.

**Keywords:** ALPPS, ischemia, laparoscopy, liver, technique

## Abstract

**Background:**

ALPPS (Associating Liver Partition and Portal vein ligation for Staged hepatectomy) is a new surgical approach for the treatment of liver tumors. It is indicated in cases where the future liver remnant is not sufficient to maintain postoperative liver function. We report a totally laparoscopic ALPPS with selective hepatic artery clamping. Pneumoperitoneum itself results in up to 53% of portal vein flow and selective hepatic artery clamping can reduce blood loss while maintaining hepatocellular function. Therefore, the combination of both techniques may result in effective control of bleeding with no damage in the liver function that may have direct impact in the result of ALPPS procedure.

**Methods:**

A 65-year-old man with colorectal liver metastases in all liver segments, except liver segment 1 (S1), were evaluated as unresectable. He underwent chemotherapy with objective response and multidisciplinary board decided for ALPPS procedure. First stage was performed entirely by laparoscopy and consisted of enucleation of metastases from segments 2 and 3, ligation of the right portal vein and liver splitting under selective common hepatic artery clamping. The second stage was done 3 weeks later and consisted of laparoscopic right trisectionectomy by laparoscopy.

**Results:**

Operative time was 250 and 200 minutes, respectively. Estimated blood loss was 150 and 100 mL. There was no need for transfusion or hospitalization in intensive care. He was discharged on the 3rd and 5th postoperative day, respectively. Recovery was uneventful after both stages and patient did not present any sign of liver failure. Elevation of liver enzymes was minimal. Computerized tomography (CT) scan before second stage showed a liver hypertrophy of 53%, sFLR was 0.37 before second stage, or 33% of the total liver volume. CT scan shows no residual liver disease and optimum liver regeneration. Patient is well with no evidence of the disease 11 months after the procedure.

**Conclusions:**

Totally laparoscopic ALPPS is a feasible and safe approach for selected patients with liver tumors. The hypertrophy of the remaining liver was adequate and sequential procedures were performed without morbidity and no mortality. Selective hepatic artery clamping seems to be an interesting solution to decrease intraoperative blood loss without the harsh effect of Pringle maneuver.

## 1 Introduction

The ALPPS procedure, which stands for Associating Liver Partition and Portal vein ligation for Staged hepatectomy, has become a new strategy for patients with otherwise nonresectable liver tumors.^[1,2]^ ALPPS consists in transection of the liver parenchyma added to the ligation of the portal vein in stage 1 of a 2-stage hepatectomy. ALPPS allows an approximately 20% increase of the entire liver volume within 1 week, achieving a future liver remnant (FLR) volume increase of 80%,^[3]^ a near doubling of the volume of a small remnant within a short period of time. However, the risk of morbidity and mortality remains high despite the observed increase in liver volumes. The main reason for this is the surgical severity of ALPPS. New modifications have been introduced to the procedure by reducing the surgical trauma during the first stage and thereby demonstrated improved safety, while maintaining the beneficial aspect of volume increase.

In our opinion, the best way to reduce surgical severity in liver surgery remains the minimal invasive approach. Pneumoperitoneum reduces the systemic inflammatory response for all types of elective surgery including liver resection.^[4,5]^ Another way to reduce postoperative liver failure and therefore liver-related morbidity is to decrease liver ischemia that is often required to avoid blood loss.

**Figure 1:**
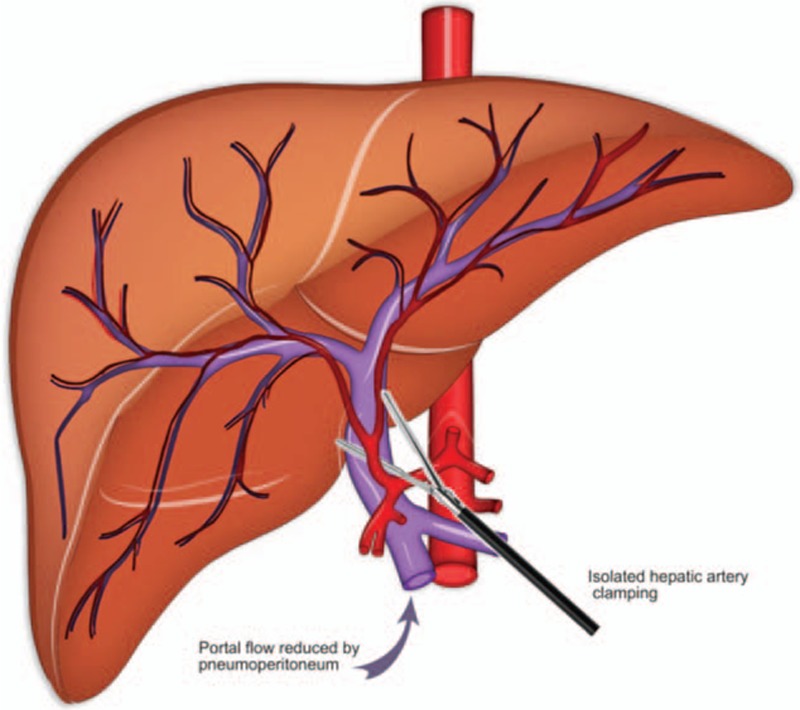
Schematic drawing of selective common hepatic artery clamping during laparoscopic ALPPS.

We report herein a totally laparoscopic ALPPS with selective hepatic artery clamping (Fig. 1). It is known that pneumoperitoneum itself results in up to 53% of portal vein flow^[6,7]^ and selective hepatic artery clamping can reduce blood loss while maintaining hepatocellular function.^[8]^ Therefore, the combination of both techniques may result in effective control of bleeding with no damage in liver function that may impact directly in the result of ALPPS procedure.

## 2 Methods

A 65-year-old man with multiple and bilateral colorectal metastases are referred for treatment after laparoscopic retosigmoidectomy. Initial evaluation with computerized tomography (CT) scan showed multiple liver metastases occupying almost all segments, except segment 1. Multidisciplinary team decided for neoadjuvant chemotherapy followed by ALPPS procedure if objective response is achieved. Informed consent was obtained. He underwent 4 cycles of chemotherapy regimen using folinic acid, fluorouracil, and irinotecan (FOLFIRI). CT scan showed reduction of liver metastasis and ALPPS procedure was proposed. The plan was to perform multiple enucleations in segments 2 and 3, followed by right portal vein ligature and in situ liver partitioning at the level of falciform ligament as stage 1. FLR (segment 1, 2, and 3) volume was estimated in 21%. Standardized future liver volume (sFLR) was 0.26. Second stage will consist in a right trisectionectomy. Both stages to be totally performed by laparoscopy with selective common hepatic artery clamping (Fig. 1). Approval to perform ALPPS was obtained and consent was obtained from patient.

**Figure 2:**
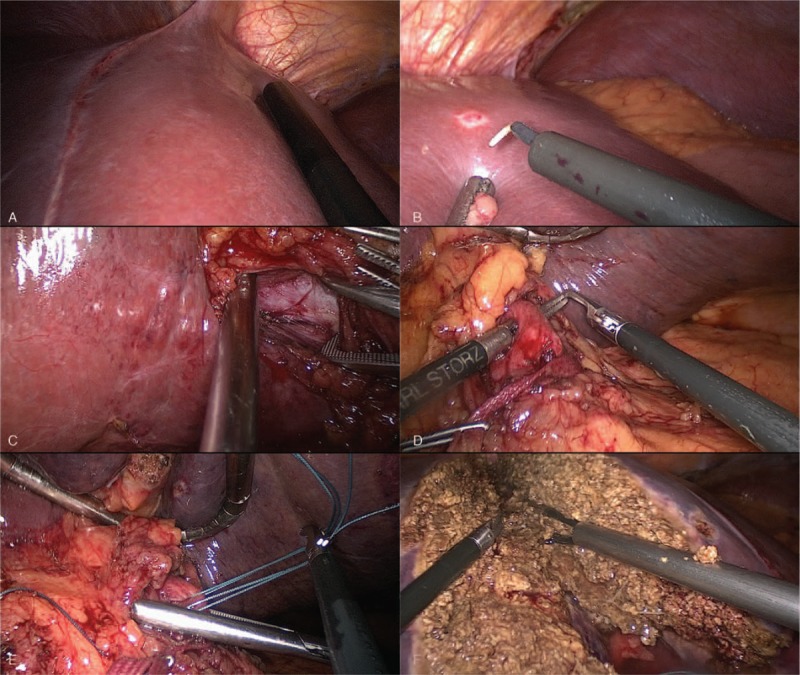
Main steps for totally laparoscopic ALPPS using selective hepatic artery clamping. (A) Intraoperative ultrasound is routinely performed and showed no new lesions. (B) Liver metastases from segments 2 and 3 were resected with free margins. (C) Right portal vein is identified. (D) Common hepatic artery is dissected and encircled. (E) Selective common hepatic artery clamping is performed. (F) Liver is partitioned with minimal blood loss.

**Figure 3:**
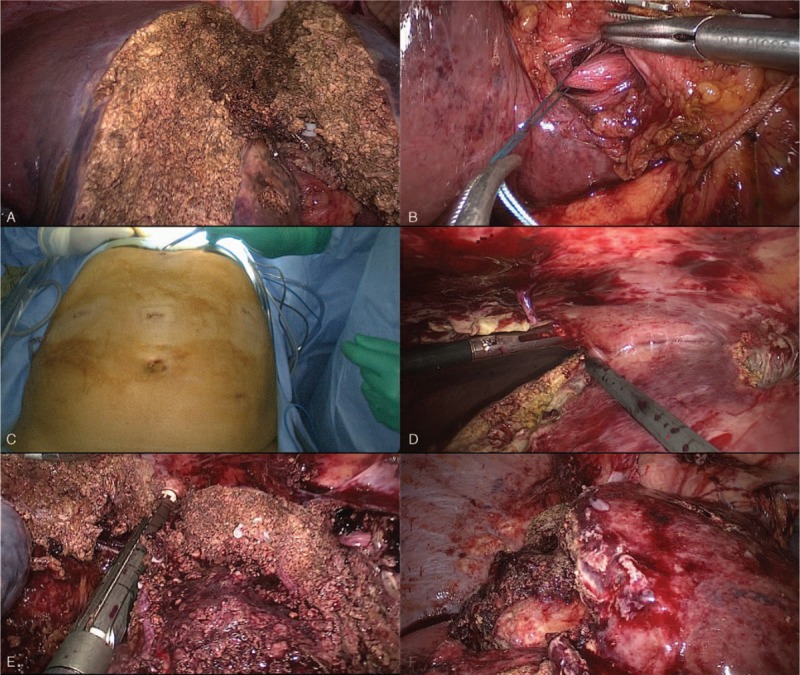
Main steps for totally laparoscopic ALPPS using selective hepatic artery clamping. (A) Liver partition is completed. (B) Right portal vein is ligated. (C) Second stage is also performed by laparoscopy and same trocar incisions from first stage were used. (D) Area of liver partition was separated with blunt maneuver. (E) Middle and right hepatic vein are divided with vascular endoscopic stapler. (F) Totally laparoscopic ALPPS is completed.

The patient is placed in a supine position with the surgeon standing between patient's legs. Pneumoperitoneum is established at a pressure of 14 mm Hg. This technique uses 5 trocars. Type and location of trocars are described elsewhere.^[9]^ At laparoscopy no peritoneal implants were detected. Intraoperative ultrasound showed no new lesions (Fig. 2A). Four superficial liver metastases were found in segments 2 and 3. Multiple enucleations were performed with free margins (Fig. 2B). Next step was to perform cholecystectomy followed by dissection of hepatic hilum with identification of the right portal vein (Fig. 2C) and common hepatic artery. Common hepatic artery is encircled (Fig. 2D). Liver is then partitioned along falciform ligament under continuous selective common hepatic artery clamping (Fig. 2E and F). After completion of liver partitioning (Fig. 3A), right portal vein is ligated (Fig. 3B). Raw surface area is covered with hemostatic tissue and a close suction drain is left along liver partition.

Patient is then discharged between stages. CT scan before second stage showed a good regeneration with adequate hypertrophy of the FLR. Second stage took place 3 weeks after first stage and consisted in a right trisectionectomy using Glissonian approach. We use the same trocar incisions (Fig. 3C). At laparoscopy there were some loose adhesions that were easily divided. Area of liver partition was separated with blunt maneuver (Fig. 3D). Right Glissonian pedicle is then divided with stapler, followed by division of middle and right hepatic vein with vascular endoscopic stapler (Fig. 3E). Surgical specimen is removed through suprapubic incision inside plastic retrieval bag. Pneumoperitoneum is reestablished and liver raw surface is reviewed for bleeding and bile leaks (Fig. 3F). Hemostatic tissue is applied and abdominal cavity is drained with closed suction drain.

## 3 Results

Operative time was 250 and 200 minutes, respectively. Estimated blood loss was 150 and 100 mL. There was no need for transfusion or hospitalization in intensive care. He was discharged on the 3rd and 5th postoperative day, respectively. Recovery was uneventful after both stages and patient did not present any sign of liver failure. Elevation of liver enzymes was minimal. CT scan before second stage showed a liver hypertrophy of 53%, sFLR was 0.37 before second stage, or 33% of the total liver volume. CT scan shows no residual liver disease and optimum liver regeneration. Patient is well with no evidence of the disease 11 months after the procedure.

## 4 Discussion

Laparoscopic technique is not oncologically inferior to open approach given that it follows the same principles of the oncologic surgery for the treatment of colorectal liver metastases. Although it lacks tactile feedback, laparoscopy allows a more extensive visual exploration of the entire abdomen than through an open incision.

ALPPS is a ligation of the portal vein plus a transection of the liver parenchyma in a first stage followed by removal of the liver in a second stage after 1 to 2 weeks and it has attracted a lot of attention because the hypertrophy induced by ALPPS is more rapid, a near doubling of the volume of a small remnant within a week.^[1,2]^ However it also has prohibitive morbidity and mortality for many groups of patients.^[10]^ Recently surgeons have modified the procedure by reduction of surgical trauma during the first stage and demonstrate an acceptable safety profile for their modifications.^[11]^ Another published modification was a partial division of the liver, “partial ALPPS,” with reported improved safety.^[12,13]^ Their rationale is a reduced surgical trauma in the first stage with similar FLR hypertrophy.

However, in our opinion, the best way to reduce surgical trauma in abdominal and liver surgery is the laparoscopic approach. Laparoscopy reduces the systemic inflammatory response for all types of elective surgery including liver resection and ALPPS procedure.^[4,5]^ On the basis of our previous experience with laparoscopic extended hepatectomies^[4]^ and staged laparoscopic hepatectomies using portal vein ligation,^[14]^ we safely performed and reported a totally laparoscopic ALPPS procedure in 2012.^[15]^ Since then, 2 other groups have reported case reports of laparoscopic ALPPS as well.^[16,17]^

It is important to note that, in nonexperienced hands, liver ischemic time can be superior in laparoscopic approach if routine use of Pringle maneuver is applied. In our experience, Pringle maneuver was not employed on a regular basis.^[18]^

Since its introduction in 2011, there was an increased worldwide experience with ALPPS procedure. The international ALPPS registry was initiated in 2012 to systematically and uniformly collect information from multiple centers around the world. The ALPPS registry enabled surgeons to study a larger population to overcome shortcomings inherent to small case series reports. Furthermore, the registry helped define the optimal patients’ selection for this complex procedure.^[3]^ The main conclusion of this study was that patients with colorectal liver metastases were the best indication for ALPPS procedure. However, patients over 60 years old, longer operations (more than 5 hours) and blood transfusions were found as risk factors for increased perioperative morbidity and mortality.

As the laparoscopic procedures became more common, studies on the effect of the pneumoperitoneum on hepatic hemodynamics were performed. The most impressive finding was that the installation of a CO_2_ pneumoperitoneum and the resulting elevation of intraabdominal pressure lead to a linear decrease of portal venous flow.^[19]^ At pressures higher than 10 mm Hg, portal flow can be significantly reduced and at 14 mm Hg portal flow may decrease up to 53%.^[6,7]^ Regarding the hepatic artery flow, several studies showed that it is not significantly modified by the increase in the intraabdominal pressure caused by pneumoperitoneum.^[20]^ So, during CO_2_ pneumoperitoneum, there is a loss of the physiologic hepatic blood flow control (hepatic arterial buffer response), that normally leads to an increase in the hepatic arterial flow when portal flow is reduced.^[21]^ Since the Pringle maneuver (developed to diminish hepatic bleeding during parenchymal transection by blocking all vascular inflow to the liver) has the detrimental effect of inducing warm hepatic ischemia that can result in postoperative liver failure and the portal flow is significantly reduced during CO_2_ pneumoperitoneum, a selective clamping of the hepatic artery can have the same beneficial effect of diminishing blood loss during hepatic transection while preserving hepatocellular function. This finding was confirmed by an experimental study using a murine model.^[8]^

The present case, according to the ALPPS registry study,^[3]^ had a higher risk for ALPPS procedure that could be partially overcome if there was no bleeding, liver failure, and operative time was less than 5 hours. In this situation, a totally laparoscopic ALPPS associated with selective hepatic artery clamping was an interesting strategy to decrease intraoperative bleeding while maintaining liver function with reduced systemic inflammatory response. Indeed, our patient had an excellent outcome and is disease free after 11 months.

## 5 Conclusions

Totally laparoscopic ALPPS is a feasible and safe approach for selected patients with liver tumors. The hypertrophy of the remaining liver was adequate and sequential procedures were performed without morbidity and no mortality. Selective hepatic artery clamping seems to be an interesting solution to decrease intraoperative blood loss without the harsh effect of Pringle maneuver.
